# Hydrogen production by Cyanobacteria

**DOI:** 10.1186/1475-2859-4-36

**Published:** 2005-12-21

**Authors:** Debajyoti Dutta, Debojyoti De, Surabhi Chaudhuri, Sanjoy K Bhattacharya

**Affiliations:** 1 Department of Biotechnology, Haldia Institute of Technology, Haldia, West Bengal, India; 2 Department of Ophthalmic Research, Cleveland Clinic Foundation, Area I31, 9500 Euclid Avenue, Cleveland, Ohio, 44195, USA

## Abstract

The limited fossil fuel prompts the prospecting of various unconventional energy sources to take over the traditional fossil fuel energy source. In this respect the use of hydrogen gas is an attractive alternate source. Attributed by its numerous advantages including those of environmentally clean, efficiency and renew ability, hydrogen gas is considered to be one of the most desired alternate. Cyanobacteria are highly promising microorganism for hydrogen production. In comparison to the traditional ways of hydrogen production (chemical, photoelectrical), Cyanobacterial hydrogen production is commercially viable. This review highlights the basic biology of cynobacterial hydrogen production, strains involved, large-scale hydrogen production and its future prospects. While integrating the existing knowledge and technology, much future improvement and progress is to be done before hydrogen is accepted as a commercial primary energy source.

## Review

Molecular hydrogen is one of the potential future energy sources as an alternative to the limited fossil fuel resources of today. Its advantages as fuel are numerous: it is eco-friendly, efficient, renewable, and during its production and utilization no CO_2 _and at most only small amounts of NO_x _are generated [1]. By the virtue of all these attributes the hydrogen gas can be used as an energy source. Hydrogen gas can be prepared in many conventional ways (including those of photoelectrochemical or thermochemical processes) for its large-scale utilization. In this review we aim to discuss about photobiological hydrogen production by cyanobacteria and the scientific and technical aspects of large-scale utilization of produced hydrogen for various applications. We also have described about salient features of cyanobacterial enzymatic system, different species and strains producing hydrogen, parameters controlling the hydrogen production and large-scale production utilizing photobioreactors. Cyanobacteria are thought to play a crucial role in the Precambrian phase by contributing oxygen to the atmosphere [2]. Under certain conditions the cyanobacterial species instead of reducing CO_2_, consume biochemical energy to produce molecular hydrogen.

### Hydrogen yielding species of cyanobacteria

Cyanobacteria form a large and diverse group of oxygenic photoautotrophic prokaryotes, many of which have the ability to produce hydrogen (Table 1). Hydrogen production has been studied in a very wide variety of cyanobacterial species and strains. Hydrogen production occurs within at least 14 Cyanobacteria genera, under a vast range of culture conditions [3]. Although a complete description of all species and their taxonomic details are beyond the scope of this review but some of them deserve special mention. Unicellular non-diazotrophic Cyanobacteria *Gloeocapsa alpicola *under sulphur starvation shows increased hydrogen production [4]. *Arthrospira *(*Spirulina platensis*) can produce hydrogen (1 μmole H_2_/12 hr/mg cell dry weight) in complete anaerobic and dark condition [5]. Another nitrogen-fixing cyanobacterium, *Anabaena cylindrica*, produces hydrogen and oxygen gas simultaneously in an argon atmosphere for 30 days in light limited condition [6]. Symbiotic Cyanobacteria within coralloid roots of the cycads *Cycas revoluta *(king Sago palm) and *Zamia furfuracea *showed a significant *in vivo *hydrogen uptake [7]. *Anabaena *sp. is able to produce significant amount of hydrogen. Among them nitrogen-starved cells of *Anabaena cylindrica *produces highest amount of hydrogen (30 ml of H_2_/lit culture/hour). Hydrogenase-deficient cyanobacteria *Nostoc punctiforme *NHM5 when incubated under high light for a long time, until the culture was depleted of CO_2 _shows increase in hydrogen production [8].

**Table 1:** Organism that produces hydrogen

**Organism name**	**Organism description**	**Maximum Hydrogen evolution**	**Growth condition**	**H_2 _evolution** ** assay condition**	**Reference**
*Anabaena cylindrica *B-629	Marine cyanobacteria	0.103 μmol/mg dry wt/h	Air contained 5% CO_2_; 7000 lx at the surface of culture vessels	Argon environment with 3% CO_2 _4000 lx at the surface of the culture vessel	9
*Oscillatoria brevis B-1567*	Marine cyanobacteria	0.168 μmol/mg dry wt/h	Air contained 5% CO_2_; 7000 lx at the surface of culture vessels	Argon environment with 3% CO_2 _4000 lx at the surface of the culture vessel	9
*Calothrix scopulorum 1410/5*	Marine cyanobacteria	0.128 μmol/mg dry wt/h	Air contained 5% CO_2_; 7000 lx at the surface of culture vessels	Argon environment with 3% CO_2 _4000 lx at the surface of the culture vessel	9
*Calothrix membrnacea B-379*	Marine cyanobacteria	0.108 μmol/mg dry wt/h	Air contained 5% CO_2_; 7000 lx at the surface of culture vessels	Argon environment with 3% CO_2 _4000 lx at the surface of the culture vessel	9
*Oscillatoria *sp. Miami BG7	Marine cyanobacteria	0.250 μmol/mg dry wt/h	Air; 100 μE/m^2^/s; NH_4_Cl (25 mg/l) used as combined nitrogen source	Ar (100%); 90 μE/m^2 ^11 day old cells 37°C	10
*Oscillatoria limosa*	Marine cyanobacteria	0.83 μmol/mg chl a/h	Air; incubation in 16 h light, 8 h darkness cycles	Same as culture condition	11
*Cyanothece *7822	Marine unicellular cyanobacteria	0.92 μmol/mg chl a/h	N_2 _with 5% CO_2_	Same as culture condition	12
*Anabaena *sp. PCC 7120	Heterocystous cyanobacteria	2.6 μmol/mg chl a/h	Air; 20 μE/m^2^/s	Air; 60 μE/m^2^/s	13
*Anabaena cylindrica *IAMM-1	Heterocystous cyanobacteria	2.1 μmol/mg chl a/h	Air; 20 μE/m^2^/s	Air; 60 μE/m^2^/s	13
*Anabaena variabilis *IAMM-58	Heterocystous cyanobacteria	4.2 μmol/mg chl a/h	Air; 20 μE/m^2^/s	Air; 60 μE/m^2^/s	13
*Anabaena cylindrica *UTEX B 629	Heterocystous cyanobacteria	0.91 μmol/mg chl a/h	Air; 20 μE/m^2^/s	Air; 60 μE/m^2^/s	13
*Anabaena flos-aquae *UTEX 1444	Heterocystous cyanobacteria	1.7 μmol/mg chl a/h	Air; 20 μE/m^2^/s	Air; 60 μE/m^2^/s	13
*Anabaena flos-aquae *UTEX LB 2558	Heterocystous cyanobacteria	3.2 μmol/mg chl a/h	Air; 20 μE/m^2^/s	Air; 60 μE/m^2^/s	13
*Anabaenopsis circularis *IAM M-13	Heterocystous cyanobacteria	0.31 μmol/mg chl a/h	Air; 20 μE/m^2^/s	Air; 60 μE/m^2^/s	13
*Nostoc muscorum *IAM M-14	Heterocystous cyanobacteria	0.60 μmol/mg chl a/h	Air; 20 μE/m^2^/s	Air; 60 μE/m^2^/s	13
*Nostoc linckia *IAM M-30	Heterocystous cyanobacteria	0.17 μmol/mg chl a/h	Air; 20 μE/m^2^/s	Air; 60 μE/m^2^/s	13
*Nostoc commune *IAM M-13	Heterocystous cyanobacteria	0.25 μmol/mg chl a/h	Air; 20 μE/m^2^/s	Air; 60 μE/m^2^/s	13
*Anabaena variabilis *AVM13	Heterocyst filamentous	68 μmol/mg chl a/h	Air and 1% CO_2_; 100 μE/m^2^/s		14
*Anabaena variabilis *PK84	Heterocyst filamentous	32.3 μmol/mg chl a/h	Air and 2% CO_2_; continuous tarbidostat mode; 113 μE/m^2^/s	Argon environment.	15
*Anabaena variabilis *PK84	Heterocyst filamentous	167.6 μmol/mg chl a/h	73%Ar, 25%N_2_, 2% CO_2_; 90 μE/m^2^/s	93%Ar, 5%N_2_, 2% CO_2_; 90 μE/m^2^/s	16
*Anabaena variabilis *PK84	Heterocyst filamentous	0.11 μmol/mg chl a/h	Air and 2% CO_2_; outdoor condition	Air and 2% CO_2_; outdoor condition; 400 W/m^2^	17
*Anabaena variabilis *ATCC 29413	Heterocyst filamentous	45.16 μmol/mg chl a/h	73%Ar, 25%N_2_, 2% CO_2_; 90 μE/m^2^/s	93%Ar, 5%N_2_, 2% CO_2_; 90 μE/m^2^/s	16
*Anabaena variabilis *ATCC 29413	Heterocyst filamentous	0.05 μmol/mg dry wt/h	5000 lx at the surface of culture vessels.	Ar and 5% CO_2_; 5000 lx addition of Tween 85 (77 mM)	18
*Anabaena variabilis *ATCC 29413	Heterocyst filamentous	39.4 μmol/mg chl a/h	Air and 2% CO_2_; continuous tarbidostat mode; 113 μE/m^2^/s	Argon environment	15
*Anabaena variabilis *1403/4B	Heterocyst filamentous	20 μmol/mg chl a/h	Air; 15 μE/m^2^/s	No gas phase; cells immobilized in hollow fiber; 25 μE/m^2^/s on the top surface and 13 μE/m^2^/s on bottom surface of reactor	19
*Anabaena azollae*	Heterocyst filamentous	38.5 μmol/mg chl a/h	Air and 1% CO_2_; 100 μE/m^2^/s	Argon environment.	16
*Anabaena variabilis *PK17R	Heterocyst filamentous	59.18 μmol/mg chl a/h	73%Ar, 25%N_2_, 2% CO_2_; 90 μE/m^2^/s	93%Ar, 5%N_2_, 2% CO_2_; 90 μE/m^2^/s	16
*Anabaena variabilis *SPU 003	Heterocyst filamentous cyanobacteria	5.58 nmol/mg dry wt/h	Air; incubation in 16 h light (3000 lx light intensity), 8 h darkness cycles; mannose used as carbon source.	Same as culture condition	20
*Synechococcus *PCC 6830	Non-nitrogen-fixing unicellular cyanobacteria	0.26 μmol/mg chl a/h	Air; photon fluence rate 20 μE/m^2^/s	Ar with CO (13.4 μmol), C_2_H_2 _ (1.34 μmol); darkness.	21
*Synechococcus *PCC 602	Non-nitrogen-fixing unicellular cyanobacteria	0.66 μmol/mg chl a/h	Air; photon fluence rate 20 μE/m^2^/s	Ar with CO (13.4 μmol); photon fluence rate was 20–30 μE/m^2^/s	21
*Synechococcus *PCC 6307	Non-nitrogen-fixing unicellular cyanobacteria	0.02 μmol/mg chl a/h	Air; photon fluence rate 20 μE/m^2^/s	Ar (100%) with photon fluence rate 20–30 μE/m^2^/s	21
*Synechoccus *PCC 6301	Non-nitrogen-fixing unicellular cyanobacteria	0.09 μmol/mg chl a/h	Air; photon fluence rate 20 μE/m^2^/s	Ar with C_2_H_2 _(1.34 μmol); fluence rate was 20–30 μE/m^2^/s	21
*Microcystis *PCC 7820	non-nitrogen-fixing unicellular cyanobacteria	0.16 μmol/mg chl a/h	Air; photon fluence rate 20 μE/m^2^/s	Ar with CO (13.4 μmol), C_2_H_2 _(1.34 μmol); photon fluence rate was 20–30 μE/m^2^/s	20
*Gloebacter *PCC 7421	Non-nitrogen-fixing unicellular cyanobacteria	1.38 μmol/mg chl a/h	Air; photon fluence rate 20 μE/m^2^/s	Ar with CO (13.4 μmol), C_2_H_2 _(1.34 μmol); photon fluence rate was 20–30 μE/m^2^/s	20
*Synechocystis *PCC 6308	Non-nitrogen-fixing unicellular cyanobacteria	0.13 μmol/mg chl a/h	Air; photon fluence rate 20 μE/m^2^/s	Ar with CO (13.4 μmol), C_2_H_2 _(1.34 μmol); photon fluence rate was 20–30 μE/m^2^/s	21
*Synechocystis *PCC 6714	Non-nitrogen-fixing unicellular cyanobacteria	0.07 μmol/mg chl a/h	Air; photon fluence rate 20 μE/m^2^/s	Ar with CO (13.4 μmol); photon fluence rate was 20–30 μE/m^2^/s	21
*Aphanocapsa montana*	Non-nitrogen-fixing unicellular cyanobacteria	0.40 μmol/mg chl a/h	Air; photon fluence rate 20 μE/m^2^/s	Ar (100%); photon fluence rate was 20–30 μE/m^2^/s	21
*Gloeocapsa alpicola *CALU 743	Unicellular non-diazotrophic cyanobacteria	0.58 μmol/mg protein	Sulphur free 4% CO_2_; 25 μmol photons/m^2^/s	Same as culture condition	4
*Chroococcidiopsis thermalis *CALU 758	Unicellular non-nitrogen-fixing	0.7 μmol/mg chl a/h	Ar and1% CO_2_; during 20 h.	Same as culture condition	22
*Mycrocystis *PCC 7806	Unicellular/colony embedded in matrix	11.3 nmol/mg prot/h	Air; incubation in 16 h light, 8 h darkness cycles	Same as culture condition	23
*Microcoleus chthonoplasts*	Mat-building cyanobacteria	1.7 nmol/mg prot/h	Air; ferric ammonium citrate added (46 μmol) 30 μE/m^2^/s	Same as culture condition	20

### Enzyme systems for hydrogen production

Cyanobacteria are photoautotrophic microorganisms [9-23] that use two sets of enzymes to generate hydrogen gas (Table 1). The first one is **Nitrogenase **and it is found in the heterocysts of filamentous cyanobacteria when they grow under nitrogen limiting conditions. Hydrogen is produced as a byproduct of fixation of nitrogen into ammonia. The reaction consumes ATP and has the general form:



A Nitrogenase enzyme consists of two parts:one is dinitrogenase (MoFe Protein, encoded by the genes *nifD *and *nifK*, α and β respectively) and the other is dinitrogenase reductase (Fe Protein, encoded by *nifH*). Dinitrogenase is a α_2_β_2 _heterotetramer, having molecular weight of about 220 to 240 kDa respectively, breaks apart the atoms of nitrogen. Dinitrogenase reductase is a homodimer of about 60 to 70 kDa and plays the specific role of mediating the transfer of electrons from the external electron donor (a ferredoxin or a flavodoxin) to the dinitrogenase [25-27]. There are three types of dinitrogenase found in Nitrogenase, which vary depending on the metal content. Type one contains molybdenum (Mo) [30], type two contains vanadium (V) instead of Mo [29,30], and type three has neither Mo nor V but it contains iron (Fe) [31,32].

The other hydrogen-metabolizing/producing enzymes in cyanobacteria are **Hydrogenases**; they occur as two distinct types in different cyanobacterial species. One type of them, uptake hydrogenase (encoded by *hupSL*) [33], has the ability to oxidize hydrogen and the other type of hydrogenase is reversible or bidirectional hydrogenase (encoded by *hoxFUYH*) and it can either take up or produce hydrogen. **Uptake hydrogenase **enzymes are found in the thylakoid membrane of heterocysts from filamentous cyanobacteria, where it transfers the electrons from hydrogen for the reduction of oxygen via the respiratory chain in a reaction known as oxyhydrogenation or Knallgas reaction. The enzyme consists of two subunits. The *hupL*-coded protein is responsible for the up taking hydrogen and the smaller subunit that is coded by *hupS *looks after the reduction affair. The hydrogen formed is usually re oxidized by an uptake hydrogenase via a Knallgas reaction and hence there is no net H_2 _production in strains with uptake hydrogenases under ambient conditions. So it is counterproductive when the goal is to produce hydrogen on a commercial scale. The reaction catalyzed by the uptake hydrogenase takes the following form:



The biological role of **bidirectional or reversible hydrogenase**, is poorly understood and thought to control ion levels in the organism. Reversible hydrogenase is associated with the cytoplasmic membrane and likely functions as an electron acceptor from both NADH and H_2 _[34]. The reversible hydrogenase is a multimeric enzyme consisting of either four or five different subunits apparently depending on the species [34,35]. Molecularly it is a [NiFe]-hydrogenase of the NAD(P)^+ ^reducing type consisting of a hydrogenase dimmer coded by *hoxYH *gene. Maturation of reversible hydrogenases requires the action of several auxillary proteins collectively termed as hyp (products of genes: *hypF*, *hypC*, *hypD*, *hypE*, *hypA*, and *hypB*) [36]. Unlike uptake hydrogenase, reversible hydrogenases are helpful in hydrogen production.

Hydrogen photo evolution catalyzed by nitrogenases or hydrogenases can only function under anaerobic conditions due to their extreme sensitivity to oxygen. Since oxygen is a byproduct of photosynthesis, organisms have developed the following spatial and temporal strategies to protect the enzyme from inactivation by oxygen, these are:

(a) Heterocyst-containing cyanobacteria physically separate oxygen evolution from nitrogenase activity by segregating oxygenic photosynthetic activity in vegetative cells and nitrogenase activity in heterocystis with reduced oxygen-permeability [37] and

(b) Non-heterocystous cyanobacteria separate oxygen-evolution from nitrogenase activity by performing these functions during light and dark periods respectively [38].

Important parameters for hydrogen production: Efficiency of hydrogen production depends on several factors or parameters that are more critical when hydrogen production is aimed at a large scale.

Various parameters influence hydrogen production in different ways, a few selected parameters are presented with examples:

A. Environmental conditions: Several environmental conditions such as light, temperature, salinity, nutrient availability, gaseous atmosphere play a role in hydrogen production. Requirement of different cyanobacterial species are different for optimum hydrogen production.

I) Light: Although most cyanobacterial species preferentially absorb red light near 680 nm [39], light requirement for hydrogen production varies among different species of cyanobacteria. While Spirulina (*Arthrospira platensis*) produces hydrogen under anaerobic conditions, both in the dark and in the light [5] but several other species produces hydrogen only in the presence of light [40]. Hydrogen production mediated by native hydrogenases in *Synechococcus *PCC7942 occurs under in the dark under anaerobic condition [41]. *Spirulina platensis *can produce hydrogen optimally at 32°C in complete anaerobic and dark condition [5]. The highest volumetric hydrogen production was found in *Anabaena variabilis *ATCC 29413 and in its mutant PK84 [42]. Hydrogen evolution by *Anabaena variabilis *PK84 with air containing 2% CO_2 _was stimulated by light [42]. Hydrogen production in *Nostoc muscorum *is catalyzed by nitrogenase more hydrogen is produced in this strain in the light than in the dark [43]. *Anabaena cylindrica*, produces hydrogen under an argon atmosphere for 30 days in light limited (luminous intensity 6.0 W/m^-2^) and 18 days under elevated light (luminous intensity 32 W/m^-2^) [6,44]. Continuous hydrogen production by *Anabaena cylindrica *for a prolonged period under light limited condition occurs in the absence of exogenous nitrogen [44]. The effect of light on nitrogenase mediated hydrogen production by most cyanobacteria is well studied [40]. Nitrogenase function is saturated only at much higher light intensities than required for optimal growth. Thus hydrogen production rates can be doubled if the luminous intensity exposure to cultures is changed from 20 W/m^2 ^to 60 W/m^2 ^[44].

II) Temperature: The optimum temperature for hydrogen production for most cyanobacterial species is between 30–40°C and varies from species to species of cyanobacteria. For example, *Nostoc *cultured at 22°C showed higher rates of hydrogen production than at 32°C [45], while *Nostoc muscorum *SPU004 showed optimum hydrogen production at 40°C [46]. *Anabaena variabilis *SPU 003 on the other hand show optimum hydrogen production at 30°C [22,23].

III) Salinity: Salinity does effect hydrogen production by cyanobacteria [43]. In general fresh water cyanobacteria shows lower rate of hydrogen production with increase in salinity. This is occurs likely because of diversion of energy and reductants for extrusion of Na^+ ^ions from within the cells or prevention of Na^+ ^influx [47].

IV) Micronutrients: Trace elements such as cobalt (Co), copper (Cu), molyblednum (Mo), zinc (Zn), and nickel (Ni) effects hydrogen production [48]. Many of these metals have shown pronounced enhancement of hydrogen production and thought to be due to their involvement in the nitrogenase enzyme. For example, *Anabaena variabilis *SPU003 is highly sensitive to Co, Cu, Mn, Zn, Ni, Fe ions and shows no hydrogen production at concentrations below 10 mM for these ions [20]. A culture of *Anabaena cylindrica *grown with 5.0 mg of Ferric ions per liter produce hydrogen at a rate about twice that of culture with 0.5 mg of Ferric ions per liter [6].

V) Carbon source: Carbon sources are also known to influence the hydrogen production considerably by influencing nitrogenase activity [46]. Presence of different carbon sources cause variation in electron donation capabilities by the cofactor compounds to nitrogenase thus influencing hydrogen production [46].

VI) Nitrogen source: Several inorganic nitrogenous compound influence hydrogen production rates in many ways. Nitrite, nitrate and ammonia have been reported to inhibit nitrogenase in *Anabaena variabilis *SPU003 and *Anabaena cylindrical *[49,46]. Generally all exogenously added nitrogen sources inhibit nitrogenase synthesis [50]. Although in *Anabaena cylindrical *ammonium addition (0.2 mM NH_4_^+^) at a given time point eventually suppresses hydrogen production, but periodic addition of lower amounts (0.1 mM ammonium chloride) do not inhibit hydrogen evolution [6]. However, influence of nitrogen source does not always part pronounced effects, and interpretation is not straightforward. While some studies showed that there are significant differences in hydrogen production depending on the nitrogen content of the media [5] while other studies show the reverse [51]. In *Anabaena cylindrica *culture, oxygen production gets dominated with the incremental addition of ammonium chloride (from 0.1 mM to 0.5 mM) [52]. The hydrogen to oxygen production ratio (4:1) in totally nitrogen starved condition decrease (1.7:1) when ammonium ions are added [52].

VII) Molecular nitrogen: Molecular nitrogen is a competitive inhibitor for hydrogen production and removal of molecular nitrogen if often very necessary for hydrogen production. Hydrogen production may be considerably inhibited in presence of molecular nitrogen [9].

VIII) Effect of oxygen on hydrogen production: Due to their extreme sensitivity to oxygen, hydrogen photo evolution catalyzed by nitrogenases or hydrogenases can function only under anaerobic conditions [37] As oxygen is a byproduct of photosynthesis, nitrogenase-containing organisms have developed several spatial and temporal separation/compartmentalization strategies as described above to protect the enzyme from inactivation by oxygenation [33].

IX) Effect of sulfur on hydrogen production: Sulfur starvation enhances the rate of hydrogen production in several cyanobacterial species (for example, *Gloeocapsa alpicola *and *Synechocystis *PCC 6803). It is possible to inhibit the oxygenic photosynthesis and enhance hydrogen production by incubating the cynobacteria in nutrients that lack sulfur [4]. Sulfur is a very important component in the photosystem II repair cycle and without sulfur the protein biosynthesis is heavily impaired and production of either cysteine or methionine becomes impossible. This results in lack of the D1 protein (32-kDa reaction center protein), essential for photosystem II and needs to be constantly replaced [4]. For these reasons during sulfur deprivation photosynthesis and respiration is decreased, even in the presence of light. Since photosynthesis decline much quicker then respiration, thus an equilibrium point is reached after a while, (usually after 22 hours) and after that the amount of oxygen that is used in respiration is greater then the oxygen released by photosynthesis and the cell become anaerobic and at this point hydrogen production occurs in higher amounts reaching peak production [4].

X) Effect of Methane: Increased hydrogen production (up to four times) is observed in *Gloeocapsa alpicola *and *Synechocystis *PCC 6803 during dark anoxic incubation when methane is present and the medium pH is between 5.0–5.5. The effect of methane on the hydrogen evolution was maximal during the first hour of the incubation followed by gradual declination [4].

B. Intrinsic factors affecting hydrogen production: There are several intrinsic factors such as genetic components or sensitive proteins in cyanobacteria that may affect hydrogen production.

I) Presence of uptake hydrogenase and decreased hydrogen yield: The net hydrogen yield is affected in strains containing uptake hydrogenase. Much of the produced hydrogen is lost due to the activity of the uptake hydrogenase [33]. Knocking out the genes coding uptake hydrogenase is therefore contemplated to result in higher hydrogen production in the species of cyanobacteria that harbor uptake hydrogenase. It is, however, also critical to over express the genes for bidirectional hydrogenase and may be achieved by transfecting cyanobacteria with plasmids containing particular genes [33].

II) Sensitivity of hydrogenase and nitrogenase to molecular oxygen: The molecular oxygen acts as an inhibitor for hydrogenase and nitrogenase. However, innovative technical interdisciplinary solutions, as described below, are now available to reduce or eliminate presence of molecular oxygen and increase yield of hydrogen [37].

a) With the advances in nanotechnology it has become possible to build semi-permeable membranes around the organisms. An example is a membrane that discriminates by size with an active transport system [53]. Nanotechnology had made possible creation of cyanobacteria with a membrane that incorporates an active transport protein system for the facilitated ejection of oxygen [53]. This system would rapidly expel the oxygen that is created during metabolism and not allow its reentry. Another membrane is the one that possesses both an oxygen-philic and oxygen-phobic sides [53]. Having the oxygen-philic side facing the bacteria and the phobic side open to the environment would facilitate the movement of oxygen away from the cells allowing the production of hydrogen to continue without being hindered by the generated oxygen.

b) Problem to oxygen sensitivity can also be addressed by using sulfur stress phenomenon to down-regulate photosynthesis as described before. This creates the anaerobic conditions required for hydrogen production. This problem may also be addressed by engineering the native hydrogenase. Engineering oxygen-tolerant hydrogenase genes, for example, *hydS *and *hydL *from *Thiocapsa roseopersicina *into sensitive organisms may help reducing the oxygen sensitivity [54]. An expression vector pEX-Tran used for Synechococcus sp. PCC7942 transformation is readily available and with minimal modification should be suitable for other cyanobacterial systems as well [54].

III) Heterocystous cyanobacteria are more efficient to produce hydrogen than cyanobacteria with vegetative cells [39,51]. These types of cyanobacteria do engage in simultaneous oxygen and hydrogen production coupled with CO_2 _fixation [51]. Problems associated with these types of Cyanobacteria are, however, high-energy requirement and separation of hydrogen and oxygen. The over expression of the genes responsible for changing vegetative state into heterocyst cell type is *hetR *(coding for the HetR protein involved in heterocyst frequency regulation, for example, in *Anabaena sp*. PCC 7120). Recombinant strains can be created with HetR protein harboring vectors that may allow with variable and controllable heterocyst frequency [39].

### Bioreactors for cyanobacterial hydrogen production

Bioreactors are essential for large-scale production of hydrogen. Since light is an essential parameter for cyanobacterial growth so all such bioreactors must be transparent and hence are called photobioreactors [41,55]. All photobioreactors require adequate entry of light, which usually is sunlight but in some photobioreactors other artificial sources of light is also used for providing controlled light. Inside photobioreactor there should be a photic zone, close to the illuminated surface and a dark zone, further away from this surface. The dark zone is due to light absorption by the cells and mutual shading. The hydrogen productivity of a photobioreactor is light limited and tends to decrease at higher light intensities (Photosynthesis diverts the hydrogen production pathway) hence the light regime is determined by the light gradient (must be diluted and distributed as much as possible; absolute dark condition responsible for highest production). Liquid circulation time or aeration (hydrogen producing enzymes are oxygen susceptible; anaerobic condition or inert gas environment is preferred) rate has something to do with hydrogen productivity. It has followed that cyanobacteria absorb preferentially red light around 680 nm. To fulfill this demand Red light panels are constructed in specialized bioreactors to provide red light to the culture systems. As a result of mixing, cells will circulate between the light and the dark zone of the reactor at a certain frequency and regular intervals, which is dependent on reactor design and gas input. The position of the light source as well as gas liquid hydrodynamics also affects cyanobacterial growth as well as hydrogen production [56].

Several types of bioreactors have been used for hydrogen production. These can be mainly divided into three types of photobioreactors (PBRs): vertical column reactor, tubular type and flat panel photobioreactor. A reactor for photobiological hydrogen production must meet several conditions:

1) Photobioreactor should be an enclosed system so that the produced hydrogen may be collected without any loss.

2) The reactor design must allow sterilization with convenience and ease.

3) To maximize the area of incident light (thus allowing high growth and hydrogen production) photobioreactor design should provide high surface to volume ratio.

(a) Vertical column reactor (air-lift loop reactor and bubble column): Such PBRs consists of a transparent column usually made up of high quality glass and surrounded by a water jacket that while allowing maintenance of the temperature with circulating water allows adequate entry of light. Reactor top has provision for medium inlet and outlets for the gases such as argon and for the hydrogen. Fresh medium is added from a reservoir from above the PBR [57,58]. Microorganisms are inoculated through a septum that helps maintenance of sterility and prevents contamination. Bottom part of the PBR column retains outlets for the culture and an inlet/outlet for argon gas. In bubble columns using sunlight as light source, the presence of gas bubbles enhances internal irradiance at sunset and sunrise. As the position of sun changes from low horizon to overhead at noon, the bubbles diminish the internal column irradiance relative to the ungassed state. The biomass productivity varies substantially during the year the peak productivity during summer may be several times greater than in the winter. An example of this type of PBR is the one used for hydrogen production using *Rhodobactor sp*. [41,57,58]. This reactor column was made up of a glass cylinder with an inner volume of 400 ml surrounded by a water jacket. The optimal dimensions of vertical column were about 0.2 m in diameter and 4 m in column height. The optimal column height depends on factors such as wind speed and strength of optically transparent materials for example, glass or thermoplastics.

(b) Flat panel Photobioreactor: A typical flat-panel PBR consists of a stainless-steel frame and three polycarbonate panels [59]. The reactor comprises of two compartments placed side by side. The front compartment contains the bacterial culture. Water is circulated via a temperature controlled water bath through the hind compartment in order to maintain the desired temperature of the culture. This design of PBR usually utilizes artificial light, tungsten-halogen lamps (usually 500 W) are placed on one side of the reactor as light source [59]. The average light intensity provided at the reactor surface is 175 W/m^2^. Alternately red light emitting diode (LED peaking at 665 nm) is used as the light source on one side [59]. A membrane gas pump circulates the gas through the spargers (hypodermic needles) at the bottom of the reactor [59]. The produced gas is collected in a gasbag. In this reactor system, pressure vessels prevent pressure fluctuations in the gas recirculation system and a pressure valve maintains a constant input pressure to the mass flow controller. A condenser prevents water vapor from entering the gas recirculation system. The reactor is autoclaved prior to cyanobacterial cultivation and hydrogen production. The culture medium is autoclaved separately and fed to the reactor. Sampling is done through the sample port, attached to the outflow tube. Bacterial growth is monitored on-line. On the right-hand side of the reactor a small tube is attached to the reactor through which bacterial suspension flows due to an airlift effect.

(c) Tubular photobioreactors: Tubular PBRs consists of long transparent tubes with diameters ranging from 3 to 6 cm, and lengths ranging from 10 to 100 m [60]. The culture liquid is pumped through these tubes by means of mechanical or airlift pumps. The tubes can be positioned in many different ways: in a horizontal plane as straight tubes with a small or large number of U-bends; vertical, coiled as a cylinder or a cone; in a vertical plane, positioned in a fence-like structure using U-bends or connected by manifolds; horizontal or inclined, parallel tubes connected by manifolds. The predominant effect of the specific designs on the light regime is a difference in the photon flux density incident on the reactor surface [61]. Tredici PBR is special type of tubular PBR with internal gas exchange and consists of thin flexible tubular plastic sleeves filled with water and ganged together with top and bottom distribution pipes [62]. The tubes are positioned in a corrugated plastic roofing sheet, which keeps them straight and even. The tubes can be of considerable length, with an optimal length in between 20 m and 50 m, depending on factor being optimized. The system is usually inclined at a slope allowing free rise of gas bubbles and a footer with compressed air line allows supply of air at the bottom of the reactor into selected tubes. The header serves as degasser to allow for containment of the fluid displaced during aeration. Every third or fourth tube is not gassed, which serves as a fluid return tube and provides an efficient airlift type of recirculation. In this PBR, cooling is achieved by water spray. The mass transfer characteristics of the tubular photobioreactor vary with the shape of the reactor and type of mixing used [63]. A summary of bioreactor types and their properties have been provided in Table 2.

**Table 2:** Summary of PBR properties

**PBR type**	**Cyanobacterial species used**	**Advantage**	**Disadvantage**	**References**
Vertical Column	*Spirulina platensi*	1. Simple and cost effective design 2. Greater rate of mass transfer in bubble columns	1. Lack of control on irradiant light. 2. Wide fluctuations in productivity.	**57**, **58**, **64**
Flat Pannel	*Spirulina platensi*	1. Greater control of incident light. 2. Effective control of gas pressure.	1. Cost for production is high. 2. Complicated design and more maintenance	**59**, **61**
Tubular	*Arthrospira platensis*, *Anabaena variabilis PK84*, *Anabaena variabilis ATCC 29413*, *Anabaena variabilis PK84*	1. Flexibility in volume to surface area ratio. 2. Flexibility in shifting the place receiving light. 3. Give higher biomass with internal static mixture.	1. While provides flexibility to irradiant light but creates pockets of poor mass transfer 2. Mixing time is longer in internal static mixture TPBR	**42**, **60**, **61**, **63**, **65**, **66**

### Future prospects for cyanobacterial hydrogen production

Cyanobacterial hydrogen production is poised to be a very useful commodity provided various effective utilization of the produced hydrogen is devised. There are various applications where the process of biological hydrogen production by cyanobacteria can be well utilized. The examples can be included from food and chemical industries, which employ the process of hydrogenation to produce derivatives that are used as food additives, commodities, and fine chemicals:

(a) Hydrogenation of cheap carbohydrates into high valued derivatives: High value-added C_5 _and C_4 _polyols can be obtained from cheap C_6 _carbohydrates by oxidative decarbonylation followed by hydrogenation. These polyols blends are useful for the manufacture of polyesters or alkyl resins employed in the manufacture of paints. Sorbitol is another important polyol produced from hydrogenation of glucose, which is used industrially in a variety of physical and chemical processes, (for example, as humectants and softener in various food products, drugs and cosmetics). Derivatives are also used in protecting coatings, plasticizers, emulsifiers and detergents.

(b) Hydrogenation of fatty acids: Hydrogenation of fatty acids is used to manufacture margarines, shortenings, and shortening oils. The hydrogenation of oils converts liquid oils into hard fats by adding hydrogen to the fat molecule. Oils can be hydrogenated to varying degrees, depending on the hardness. These fats are desirable for its melting point, allowing for high temperature cooking and frying. Hydrogenation involves the artificial saturation of unsaturated bond(s) present in fatty acids. In the process fatty acids are put under pressure, using hydrogen gas at temperatures of 120–210°C (248–410°F) in the presence of a metal catalyst (nickel, platinum, or copper) [67] for six to eight hours.

All these process requires the rigorous hydrogenation. If hydrogen produced by cyanobacteria is very economical in comparison to the traditional large-scale hydrogen production. Cyanobacterial hydrogen produced in a photobioreactor can easily be directed to separate compartments containing the substrate for hydrogenation and specific catalysts. Provisions of external heating as well as purifying the products from different contaminants (catalyst and reactants) may be easily provided as well. Hydrogen generated in near vicinity in PBRs will reduce requirement for transport and hazards associated with transport. Apart from hydrogenation, hydrogen is combustible so it can be well applied as a substitute for conventional fuel or may be used in fuel cells to generate electricity.

## Conclusion

Hydrogen gas is seen, as a future energy carrier by virtue of the fact that it does not evolve the "greenhouse gas" CO_2 _in combustion, liberates large amounts of energy per unit weight in combustion, easily converted to electricity, and is an inexhaustible resource. Biological hydrogen production has several advantages over other conventional hydrogen production processes. It requires the use of a simple photobioreactor akin to a transparent closed box, with low energy requirements and it is very cost effective. Electrochemical hydrogen production via solar battery-based water splitting requires the use of solar batteries with high-energy requirements. Low conversion efficiencies of biological systems can be compensated for, by low energy requirements and reduced initial investment costs. The most appealing aspect of the biological hydrogen production is the source of hydrogen, which is nothing but water. With the existing knowledge of bioengineering it possible to obtain sufficient amount of hydrogen that on combustion will liberate energy and therefore could act as a substitute of coal in several operations [68]. Reported analysis suggests that cost of photobiological produced hydrogen ($25/m^3^) is much lower compared to that produced by photovoltaic splitting of water ($170/m^3^) [69]. Though there are various hindrances with cyanobacterial hydrogen production and utilization but potential solutions also seem to exist readily. With the global population increasing at a fast and steady rate, both the environment and the earth's natural resources cannot continue to be exploited without development of alternative sources of energy. Capability of individual nations to produce hydrogen will eliminate monopolies on the fuel industries, and, price increases due to political conditions. The hydrogen production by cyanobacteria although offers much promise in this respect, more research is needed before this commodity can be effectively utilized.

## Abbreviations

ADP Adenosine di-phosphate; CO_2 _Carbon dioxide; ATP Adenosine triphosphate; NO_x _Nitrogen oxides; Mo Molybdenum; Fe Iron; V Vanadium; PBR Photo bioreactor; H_2 _Hydrogen; O_2 _Oxygen; N_2 _Nitrogen; NADP Nicotinamide Adenine di nucleotide Phosphate; Ni Nickel; Co Cobalt; Cu Copper; Zn Zinc; Mn Manganese; Na^+ ^Sodium ion; CH_4 _Methane; C_4 _Four carbon compounds; C_5 _Five carbon compounds; C_6 _Six-carbon compounds.

## Authors' contributions

DD and DD collected and read the papers and prepared a rough skeleton of manuscript. SC co-directed and supervised the work. SKB directed and prepared the final manuscript.
